# BDNF reverses aging-related microglial activation

**DOI:** 10.1186/s12974-020-01887-1

**Published:** 2020-07-14

**Authors:** Shih-Ying Wu, Bo-Syong Pan, Sheng-Feng Tsai, Yi-Ting Chiang, Bu-Miin Huang, Fan-E Mo, Yu-Min Kuo

**Affiliations:** 1 Institute of Basic Medical Sciences, College of Medicine, National Cheng Kung University, Tainan, Taiwan; 2 Department of Cell Biology and Anatomy, College of Medicine, National Cheng Kung University, 1 Ta Hsueh Road, 70101 Tainan, Taiwan

**Keywords:** Microglial activation, BDNF, TrkB, Aging, CREB, NF-кB

## Abstract

**Background:**

Excessive microglial activation is implicated in the pathogenesis of various age-related neurodegenerative diseases. In addition to neurons, brain-derived neurotrophic factor (BDNF) and its receptor TrkB are also expressed in microglia. However, the direct effect of BDNF on age-related microglial activation has rarely been investigated.

**Methods:**

We began to address this question by examining the effect of age on microglial activation and the BDNF-TrkB pathway in mice. By using pharmacological and genetic approaches, the roles of BDNF and downstream signaling pathways in microglial activation and related neurotoxicity were examined in microglial cell line and primary microglial cells.

**Results:**

We showed that microglial activation was evident in the brains of aged mice. The levels of BDNF and TrkB in microglia decreased with age and negatively correlated with their activation statuses in mice during aging. Interestingly, aging-related microglial activation could be reversed by chronic, subcutaneous perfusion of BDNF. Peripheral lipopolysaccharide (LPS) injection-induced microglial activation could be reduced by local supplement of BDNF, while shTrkB induced local microglial activation in naïve mice. In cultured microglial cell line and primary microglial cells, BDNF inhibited LPS-induced microglial activation, including morphological changes, activations of p38, JNK, and NF-кB, and productions of proinflammatory cytokines. These effects were blocked by shTrkB. BDNF induced activations of ErK and CREB which then competed with LPS-induced activation of NF-кB for binding to a common coactivator, CREB-binding protein.

**Conclusions:**

Decreasing BDNF-TrkB signaling during aging favors microglial activation, while upregulation BDNF signaling inhibits microglial activation via the TrkB-Erk-CREB pathway.

## Background

Microglial activation is implicated in the pathogenesis of multiple neurodegenerative diseases [1]. Under physiological conditions, microglia are in a resting state characterized by ramified morphology, and they function as homeostatic keepers of the central nervous system [2, 3]. Resting microglia are not dormant; their processes are constantly and actively scanning a defined territory of brain parenchyma [2, 3]. After they have been exposed to stimulatory signals, microglia undergo various degrees of activation, such as changing their morphology, gene expression, and functional behavior [3]. Depending upon the type, intensity, and duration of the exposure to the stimuli, activated microglia can be neuroprotective or neurotoxic [3]. Activated microglia can release various inflammatory cytokines and toxins that together might injure or even cause neuronal death [3].

Brain-derived neurotrophic factor (BDNF), a versatile member of the neurotrophin family, is widely and highly expressed in the brain and is a chief regulator of axonal growth, neuronal differentiation, survival, and synaptic plasticity [4]. In the central nervous system, BDNF and downstream prosurvival pathways have been demonstrated to protect neurons from damage and enhance neuronal network reorganization after injury [4, 5]. It has also been reported that BDNF treatment could reduce degrees of microglial activation in certain brain injury models [6–9], albeit these responses were considered a consequence of reduced neuronal injury and death elicited by BDNF. The direct effect of BDNF on microglia has rarely been explored.

This study aimed to characterize the role of BDNF in age-related microglial activation. Initially, we found that degrees of microglial activation were especially evident in the substantia nigra (SN) across different brain regions of aged mice. Because the SN has the highest microglia density in the brain and is one of the most sensitive brain regions in response to inflammatory stimulation [10–12], we then focused on the SN to examine the effect of age on microglial activation and the BDNF-TrkB pathway. By using pharmacological and genetic approaches, the roles of BDNF and downstream signaling pathways in microglial activation and related neurotoxicity were examined in microglial cell line and primary microglial cells. Our results strongly suggested a direct antimicroglial activation effect of BDNF via the TrkB-Erk-CREB signaling pathway.

## Methods

### Animals

It has been shown that the degrees of microglial activation increased with age in both genders, but were more pronounced in males, as were peripheral LPS-induced microglial activation [13]. Furthermore, sex differences in the microglia have been suggested to play a vital role in sex-related susceptibility to psychiatric and neurological disorders [14, 15]. To avoid the sex-dependent confounding effects, we only used male mice for the *in vivo* experiments.

Animals were treated in accordance with the U.S. National Institutes of Health Animal Protection Guidelines and approved by the National Cheng Kung University Institutional Animal Care and Use Committee (IACUC number 101065). Male C57BL/6 J mice (3, 6, and 12 months old) were obtained from the National Cheng Kung University’s Laboratory Animal Center (http://www.ncku.edu.tw/animal/eng/nckulac.html) and the National Laboratory Animal Center Tainan Facility (http://www.nlac.org.tw/english/default.asp); both are located in Tainan, Taiwan. The mice were housed (four to five per cage) under a 12-h light/12-h dark cycle (lights on at 8 A.M. and lights off at 8 P.M.) at a stable temperature (24 ± 1 °C) and humidity in a control room under the supervision of qualified caretakers in the Laboratory Animal Center. The mice were given free access to food and water. The number of animals used in each experiment was listed in Table 1.

**Table 1:** Groups and number of mice used for each experiment

Experiment	No. of groups	No. of animals
Age effects on microglial activation in region of interest (Fig. S1)	2 (3 and 12 months)	6 (3/group)
Expression of Iba-1, p-p65/p65, BDNF, TrkB, TNF, IL-6, TH, and DAT in the SN of mice (Figs. 1a, c, e–i, 2e–h, and 7a–b)	3 (3, 6, and 12 months)	24 (8/group)
Flow cytometry assay of number of Iba1^+^ cells in the brain of mice (Fig. 1b)	3 (3, 6, and 12 months)	9 (3/group)
Expression of MHC class II in the SN of mice (Fig. 1d)	3 (3, 6, and 12 months)	15 (5/group)
Dual immunofluorescence staining of BDNF/TrkB and Iba1 in the SN of mice (Fig. 2a–d)	2 (6 and 12 months)	6 (3/group)
Effects of subcutaneous BDNF supplementation on expression of Iba1in the SN of age mice (Fig. 3a)	3 (0, 0.1, and 1 μg of BDNF)	14 (4, 5, and 5)
Effects of intra-brain infusion of BDNF on systemic LPS-induced local microglial activation in mice (Fig. 3b and S2a)	3 (SN, striatum, and hippocampus) Each mouse received saline injection to the left hemisphere and BDNF to the right hemisphere.	12 (4/group)
Effects of knockdown of TrkB on the microglial activation in the SN, striatum, and hippocampus of mice (Figs. 3c and S2b)	3 (SN, striatum, and hippocampus) Each mouse received shLacZ virus injection to the left hemisphere and shTrkB viruses to the right hemisphere.	9 (3/group)
Primary microglial cultures (Figs. 4e–h and 7d)	not applicable	42 pups

### Microglial cell culture

Both immortalized murine microglial BV2 cells (ATCC, Manassas, VA, USA) and mesencephalic primary microglial cells were used. BV2 cells were cultured in Dulbecco’s modified Eagle’s medium/F12 (DMEM/F12) (Cat #: 11320082, Thermo Fisher Scientific, Waltham, MA, USA) containing 10% fetal bovine serum (FBS) (Cat #:10082147, Thermo Fisher Scientific) in an atmosphere containing 5% CO_2_ at 37 °C. Mesencephalic primary microglial cells were prepared as described [16]. Briefly, the brains of 1-day-old C57BL/6 mice were removed, and the mesencephalic region was dissected out in ice-cold, sterile phosphate-buffered saline (PBS; 137 mM NaCl, 2.7 mM KCl, 8 mM Na_2_HPO_4_, and 2 mM KH_2_PO_4_) that contained 18 mM of glucose (Cat #: G8270, Sigma-Aldrich, St. Louis, MO, USA) and 1% antibiotics (50 U/ml of penicillin and 50 μg/ml of streptomycin, Cat #:15140122, Thermo Fisher Scientific). The mesencephalic specimens were trypsinized for 10 min, and the cells (5 × 10^5^/cm^2^) were incubated in DMEM/F12 containing 10% FBS until they reached confluence (~ 14 day). The flask was shaken at 180 rpm for 5 h and centrifuged to collect the floating cells. The pelleted cells were suspended and cultured in DMEM/F12 containing 10% FBS and 1 mM of sodium pyruvate in a poly l-lysine (20 μg/ml, Cat #: P8920, Sigma-Aldrich) precoated 6-cm dish for 3 days to obtain enriched microglia.

To characterize the effect of BDNF on microglia, cells (10^6^) were treated with BDNF (100 ng/ml) (Cat #: 450-02, PeproTech, Rocky Hill, NJ, USA) before (Pre), concurrently (Co), and after (Post) adding LPS (10 ng/ml) to the culture media. In the Pre group, cells were first cultured for 30 min in medium containing BDNF, washed, and then cultured for 30 min in medium containing LPS, washed, and finally cultured for 30 min in fresh medium. In the Co group, cells were cultured for 30 min in medium containing BDNF and LPS, washed, and then cultured for 30 min in fresh medium. In the Post group, cells were first cultured for 30 min in medium containing LPS, washed, and then cultured for 30 min in medium containing BDNF.

To investigate the downstream signaling pathway, cells were first cultured in medium containing Erk inhibitor, PD98059 (5 μM, Cat #: 1213, Tocris Bioscience, Avonmouth, Bristol, UK), for 8 h before the addition of BDNF for 30 min. The cells were then cultured for 30 min in medium containing LPS, washed, and finally cultured for 30 min in fresh medium. The cells were collected, and Western blotting was used to quantify protein levels.

### Conditioned medium neurotoxicity assay

For neurotoxicity assay, conditioned media were collected 60 min after the end of LPS and BDNF treatments in the BV2, viral infected BV2, and primary microglial cultures. The detailed timelines are given in the respective figures (Fig. 7c–e). TH^+^ neurons were obtained from differentiated human SH-SY5Y neuroblastoma cells (ATCC) as previously described [17]. Shortly, SH-SY5Y cells were cultured for 16 h in a 96-well plate (6 × 10^3^/well) with 100 μl of DMEM/F12 per well. The culture medium was then changed to DMEM/F12 with retinoic acid (10 μM) and BDNF (10 ng/ml) to induce differentiation (Day 0) and replaced with fresh medium every 3 days. TH^+^ neurons were confirmed by dual expression of tyrosine hydroxylase (TH) and mature neuron marker, microtubule-associated protein 2. Conditioned media were added to the differentiated cells at Day 10. At Day 11, 3-[4,5-dimethylthiazol]-2,5-diphenyltetrazolium bromide (Cat. #: M2128, Sigma-Aldrich) was added to each well to make a final concentration of 0.5 mg/ml. The plate was incubated at 37 °C for 4 h before the medium was removed. Finally, 50 μl of dimethylsulfoxide was added to each well, and the plate was gently shaken in the dark for 20 min. The plate was read in an ELISA microplate reader (VersaMax; Molecular Devices, Sunnyvale, CA, USA) at an absorbance wavelength of 570 nm.

### Immunohistochemical staining

The mice were anesthetized with an overdose of isoflurane and perfused from the left ventricle with ice-cold 0.1 M PBS. Their brains were quickly removed. The right hemispheres were stored at − 80 °C for biochemical analyses. The left hemispheres were fixed in 4% paraformaldehyde in 0.1 M phosphate buffer for 2 days at 4 °C. The brain specimens were then dehydrated in graded sucrose solutions (10%, 20%, 30%, and 35%, dissolved in 0.1 M phosphate buffer) and embedded with frozen section media (Cat. #: 3801480, Leica Biosystems, Wetzlar, Hessen, Germany). The brains were coronally sliced into 30-μm sections and stored in cryoprotectant at − 20 °C. The brain sections of interest were selected, washed with phosphate-buffered saline containing 0.3% Triton X-100 to remove the embedding frozen section media, immersed in 3% H_2_O_2_ to abolish endogenous peroxidase activity, and blocked with 3% normal goat serum for 1 h at room temperature. The free-floating brain sections were stained using rabbit anti-ionized calcium-binding adapter molecule-1 (Iba1) (1:2,000, Cat. #: 019-19741, Wako Pure Chemical Industries, Osaka, Japan) for microglia, mouse anti-major histocompatibility complex (MHC) Class II (1:500, Cat. #: 68258S, Cell Signaling Technology, Danvers, MA, USA) for activated microglia, rabbit anti-tyrosine hydroxylase (1:2,000, Cat. #: MAB152, Millipore, Darmstadt, Germany) for dopaminergic (DA) neurons, and anti-dopamine transporter (DAT) (1:1,000, Cat. #: sc-1433, Santa Cruz Biotechnology, Dallas, TX, USA) for DA neuron terminals. Brain sections were then incubated with appropriate biotin-conjugated secondary antibodies and avidin-biotin peroxidase (Vectastain Elite ABC Kit; Vector Laboratories, Burlingame, CA, USA) using diaminobenzidine as the substrate. When needed, the developed signals were enhanced using nickel ammonium sulfate [18]. The signals were evaluated based on their morphologies and intensities after subtracting the signals of the primary antibody-omitted negative controls.

For dual immunofluorescence staining, adjacent 10-μm coronal sections were incubated in a PBS solution containing 0.1% Triton X-100 (Cat. #: X100, Sigma-Aldrich) and 3% bovine serum albumin (Cat. #: A7030, Sigma-Aldrich) for 1 h at room temperature and then transferred to a buffer that contained either BDNF (1:500, Cat. #: sc-65514, Santa Cruz Biotechnology) or TrkB (1:1,000, Cat. #: sc-7268 Santa Cruz Biotechnology) antibodies overnight at 4 °C. Appropriate secondary antibodies against mouse IgG, conjugated with fluorescent dye Alexa-Fluor 594 (1:1,000, Cat. #: A-11005, Thermo Fisher Scientific), were used to detect the expression of BDNF and TrkB. The sections were then incubated overnight at 4 °C in a solution containing anti-Iba1 (1:2,000, Cat. #: 019-19741, Wako Pure Chemical Industries) antibodies. Antibodies against rabbit IgG, conjugated with fluorescent dyes Alexa-Fluor 488 (1:1,000, Cat. #: A-11008, Thermo Fisher Scientific), were utilized to detect the Iba-1 antibodies. Incubations without primary antibody were used as negative controls. Images were acquired using a confocal microscope (FV1000MPE; Olympus, Tokyo, Japan) connected to a computer equipped with imaging software (FV10-ASW; Olympus). In some cases, the BDNF and TrkB antibodies were replaced by isotype antibodies to control for non-specific binding of the antibodies.

### Morphological analysis

The area of Iba1^+^ cells was measured in a fixed area or every sixth section of the entire SN (bregma − 2.54 to − 3.88 mm). Photomicrographs were taken using a digital camera (Axiocam MRc; Carl Zeiss) connected to a computer equipped with imaging software (Axiovision 4.8; Carl Zeiss). The Iba1^+^ areas were obtained using image analysis software (Image-Pro Plus 6.0; Media Cybernetics, Rockville, MD, USA) by measuring the areas with Iba1^+^ intensities higher than a given background threshold. The background intensity threshold was fixed and used for all sections.

The number of Iba1^+^ cells was counted in every sixth section on the right SN using a modified stereological procedure [10]. The two researchers who performed the cell counting using different sets of sections (i.e., set 1: section # 1, 7, 13, 19, 25, and 31; set 2: section # 2, 8, 14, 20, 26, and 32) were blinded to the treatment. The SN was first outlined under a × 10 objective lens, and the number of positively stained cells was counted using the optical dissection (1 μm per section) method employing a computer-controlled x-y-z motorized stage and × 100 oil immersion objective (Carl Zeiss). The cell counting began 2 μm below the top of the section. The number of labeled cells per section was divided by the slide selection ratio (6/36) to obtain the total number of cells in each SN.

### Flow cytometry

C57BL/6 mice (3, 6, and 12 months old) were anesthetized with an overdose of chloral hydrate and then perfused with ice-cold saline. Their brains were removed and placed in ice-cold Hank’s balanced salt solution (Invitrogen) containing 2.5 mg/ml of trypsin and dissociated using mechanical shearing. The cell suspension homogenates were passed through a 30-μm nylon membrane (Becton-Dickinson Labware, Franklin Lakes, NJ, USA) and then centrifuged at 500×*g* for 10 min. The pellets were suspended in PBS, and microglial cells were isolated using a combination of protocols of differential density (stepwise Percoll) gradient centrifugation and immunomagnetic (Iba1) cell separation [19]. Purified microglial cells were fixed in 4% paraformaldehyde at 4 °C for 1 h and then incubated with Iba1 antibody (1:250) (Wako) at 4 °C for 16 h. After they had been washed twice with PBS, the cells were incubated for 4 h with goat anti-rabbit-Alexa-Fluor 488 (1:500) (Invitrogen) antibodies. The cells were then washed two more times, and propidium iodide was added for 1 h at room temperature. The stained cells were injected into a flow cytometer (FACScan; Becton-Dickinson, Mountain View, CA, USA), and the fluorescence emission was measured at 515 nm. The percentage of cells was calculated using Cell-QuestTM software (Becton-Dickinson).

### Immunoprecipitation and Western blot

Brain tissues and cells were homogenized in RIPA buffer (1% NP40, 1 mM of phenylmethanesulfonyl fluoride, 10 μg/ml of aprotinin, 1 μg/ml of leupeptin, 0.5 mM of sodium vanadate, 137 mM of NaCl, 20 mM of Tris-HCl [pH 8.0]) supplemented with protease and phosphatase inhibitors cocktails (Cat. #: 4693132001 and 4906837001, Roche, Basel, Switzerland) at a ratio (sample mass:buffer volume) of 1:1 for brain tissues and 1:3 for cultured cells, and, centrifuged at 17,000×*g* for 30 min at 4 °C. The protein concentrations of the supernatants were determined and adjusted to the same concentration.

For immunoprecipitation, supernatants were incubated with protein A/G PLUS-Dynabeads (Cat. #: 88803, Thermo Fisher Scientific) for 3 h at 4 °C. Pre-cleared lysates were then subjected to immunoprecipitation using anti-CREB-binding protein (CBP) antibodies (Cat. #: 7389S, Cell Signaling Technology) and protein A/G PLUS-Dynabeads (Invitrogen) for 16 h at 4 °C. The proteins co-immunoprecipitated with CBP were analyzed by Western blotting.

Supernatants (30 μg of total protein) were mixed with sample buffer containing 0.5 M of dithiothreitol, heated to 80 °C for 10 min, loaded into each well of 4–12% polyacrylamide gel (Nu-PAGE gel; Invitrogen), and resolved at 120 V for 2 h.

The separated proteins were transferred to a polyvinylidene fluoride membrane (Cat. #: 1620177, Bio-Rad Laboratories, Hercules, CA, USA), blocked in 5% milk, and probed with respective primary antibodies: BDNF (1:1,000, Cat. #: sc-33905, Santa Cruz Biotechnology), TrkB (1:3,000, Cat. #: BD610102, BD Biosciences, San Jose, CA, USA,), p-38 MAPK (1:10,000, Cat. #: 9212, Cell Signaling Technology), Phospho-p-38 MAPK (Cat. #: 4092, 1:10,000, Cell Signaling Technology), JNK (1:20,000, Cat. #: 9252, Cell signaling Technology), Phospho-JNK (1:10,000, Cat. #: 9251, Cell Signaling Technology), Erk (1:20,000, Cat. #: 9102S, Cell Signaling Technology), Phospho-Erk (1:10,000, Cat. #: 9101S, Cell Signaling Technology), NF-κB p65 (1:20,000, Cat. #: 8242S, Cell Signaling Technology), Phospho-NF-κB p65 (1:20,000, Cat. #:3036S, Cell Signaling Technology), CREB-binding protein (CBP) (1:20,000, Cat. #: 7389S, Cell Signaling Technology), glycogen synthase kinase 3 (GSK3) (1:5,000, Cat. #: 5676, Cell Signaling Technology), Phospho-GSK3-β^Y216^ (1:5,000, Cat. #: 75745, Abcam, Cambridge, UK), Phospho-GSK3-β^S9^ (1:5,000, Cat. #: 9336, Cell Signaling Technology), CREB (1:10,000, Cat. #: 9197, Cell Signaling Technology), Phospho-CREB (1:20,000, Cat. #: 9198S, Cell Signaling Technology), Akt (1:20,000, Cat. #: 9272S, Cell Signaling Technology), Phospho-Akt (1:20,000, Cat. #: 9271S Cell Signaling Technology), Mitogen-activated protein kinase phosphatase-1 (MKP-1) (1:5,000, Cat. #: sc-1199, Santa Cruz Biotechnology), and β-actin (1:40,000, Cat. #: MAB1501, Merck Millipore, Burlington, MA, USA). The bound antibodies were detected using an enhanced chemiluminescence detection kit (Cat. #: NEL103001EA, PerkinElmer, Boston, MA, USA). The band densities were measured using an imaging system (BioChemi; UVP, Upland, CA, USA) and analyzed using ImageJ (1.43u) (http://rsb.info.nih.gov/ij/). For gel loading control, membranes were re-probed with monoclonal β-actin antibody (1:40,000, Cat. #: MAB1501, Merck Millipore).

### ELISA

Mouse TNF (Cat. #: BMS607-3, Thermo Fisher Scientific) and IL-6 (Cat. #: KMC0061, Thermo Fisher Scientific) ELISA kits were used to quantify the levels of TNF and IL-6 in the supernatants and conditioned medium of microglial cells. The plates were read in the ELISA microplate reader at an absorbance wavelength of 405 nm. Standard curves were obtained from known concentrations of TNF and IL-6 provided by the kits.

### BDNF supplement to mice

The 18-month-old mice were given a BDNF supplement for 1 month. The BDNF solution contained 1 μg or 0.1 μg of BDNF (Cat #: 450-02, PeproTech) dissolved in 75 μl of 0.9% saline and filled in the Alzet osmotic mini-pump (Model 1004-100 μl; Durect, Cupertino, CA, USA) at a rate of 0.11 μl/h for 28 days. The mini-pump was implanted subcutaneously, superior to the scapular. Mice implanted with 0.9% saline-filled osmotic mini-pumps were vehicle controls.

In another study, 3-month-old male mice were given an intracranial injection of BDNF 30 min before LPS injection. BDNF (1 μl, 0.1 μg in 1 μl of 0.9% saline) (Cat. #: 450-02, PeproTech) was injected into the right striatum (stereotaxic coordinates in millimeter from the bregma: anterior/posterior, + 0.14; lateral, − 2.0; ventral, − 3.5), hippocampus (mm from the bregma: anterior/posterior, − 2.3; lateral, − 1.5; ventral, − 2.1), and SN (milllimeter from the bregma: anterior/posterior, − 3.0; lateral, − 1.2; ventral, − 4.4). An equal volume of saline was injected into the left respective brain regions to create an internal sham control. The infusion was controlled using a syringe pump at a rate of 0.1 μl/min. The needle was removed 10 min after the infusion was completed. Thirty minutes later, the mice were intraperitoneally injected with 0.15 mg/kg of LPS and given an overdose of chloral hydrate 6 h after the LPS injection.

### Lentivirus production

The lentiviral plasmids (pLKO.1-puro) expressing shRNAs against TrkB and LacZ were obtained from the National RNAi Core Facility of Academia Sinica, Taipei, Taiwan. To knockdown TrkB, three plasmids expressing three different shRNA sequences were generated. Their knockdown efficiencies were examined by transfecting the shTrkB- and shLacZ-expressing plasmids into mouse neuroblastoma N2a cells, and then Western blotting was used to quantify the TrkB level. The chosen encoding sequences of shRNA against TrkB and LacZ were 5′-CAGCAACCTGCGGCACATAAA-3′ and 5′-TGTTCGCATTATCCGAACCAT-3′, respectively. The lentivirus was packaged by transiently co-transfecting shRNA expression plasmid, psPAX2 packaging plasmid, and pMD2G envelope plasmid into HEK293T cells. The medium was collected 48 h post-transfection, and the debris was removed using low-speed centrifugation. High-titer (~ 10^8^ IU/ml) stocks were prepared using ultracentrifugation at 100,000×*g* for 90 min at 4 °C. Viral pellets were suspended in a serum-free medium and stored at − 70 °C. The lentiviral titers were examined using the p24 ELISA kit (Cat. #: 632200, Takara Bio, Kyoto, Japan).

### Knockdown of TrkB

shTrkB viral solution (1 μl) was injected into the right SN (stereotaxic coordinates in mm from the bregma: anterior/posterior, − 3.0; lateral, − 1.2; ventral, − 4.4), striatum (from the bregma: anterior/posterior, + 0.14; lateral, − 2.0; ventral, − 3.5), and hippocampus (from the bregma: anterior/posterior, − 2.3; lateral, − 1.5; ventral, − 2.1) of 3-month-old mice. An equal volume of shLacZ viral solution was injected into the left relative brain regions to create an internal sham control. The infusion was controlled using a syringe pump at a rate of 0.1 μl/min. The needle was removed 10 min after the infusion was completed. The mice were killed 1 week later, and their brains were harvested for subsequent analyses.

For cell study, the shTrkB viruses were encapsulated in phosphatidylserine-containing liposomes that contained green fluorescent indicators (PS-liposomes) (Cat. #: 810198C; Avanti Polar Lipids, Alabaster, AL, USA) that increased phagocytosis by macrophages and microglia. PS-liposomes were first diluted in reduced serum medium, Opti-MEM (Cat #: 31985070, Thermo Fisher Scientific), to a concentration of 0.1%, and then incubated with shTrkB viruses (2 × 10^6^ IU/ml) for 30 min at 37 °C. For infection, BV2 cells (10^6^ in a 10-cm dish) were cultured in DMEM/F12 containing 10% FBS without antibiotics for 16 h and then replaced with Opti-MEM medium with shTrkB+PS-liposomes (0.1%) at 37 °C for 8 h. The cells were washed and cultured in DMEM/F12 medium with 10% FBS without antibiotics for 72 h before they underwent a protein analysis. The experiment was performed in triplicate. Negative control cells were transfected with shLacZ+PS-liposomes. The infection efficiency (number of cells with green fluorescent/total number of cells) was approximately 90%.

### Statistical analysis

The total number (*n*) of observations in each group is indicated in figure captions. All data were randomly collected. Statistical analyses were performed with SPSS 17.0. Data were plotted as mean ± standard deviation (S.D.). Significance was set at *p* < 0.05. The area of Iba1^+^ signals, number of Iba1^+^, concentrations of cytokines, and levels of BDNF and TrkB between different age groups were analyzed using one-way ANOVA and then Bonferroni post-hoc tests if the overall effect was significant. The Kruskal-Wallis test was used when the assumptions of data normal distribution were not met. Paired two-tailed Student’s *t* tests were used to analyze the effects of BDNF and shTrkB treatments on mice, as well as the BDNF^+^ and TrkB^+^ signals in the Iba1^+^ cells. Two-way ANOVA was used to analyze the main effects of shTrkB and any possible interaction between them. Bonferroni post-hoc tests were done if the main effects or interactions were significant.

## Results

### Levels of BDNF and TrkB are negatively associated with microglial activation during aging

The role of BDNF in microglial activation during normal aging was investigated. Initially, we characterized degrees of microglial activation across different brain regions of young (3-month-old) and aged (12-month-old) male C57BL/6 mice. Microglia were identified by immunostaining Iba1, a marker for resting and activated microglia. We defined the degrees of microglial activation by the areas (%) of the Iba1^+^ signals and the numbers of Iba1^+^ cells in the sampled sections. Among the 5 examined brain regions (cortex, hippocampus, thalamus, VTA, and SN), the SN had the largest Iba1^+^ areas of aged mice (Fig. S1). Therefore, the SN was selected as the region of interest to study the relationship between BDNF and microglial activation during aging.

**Fig. 1:**
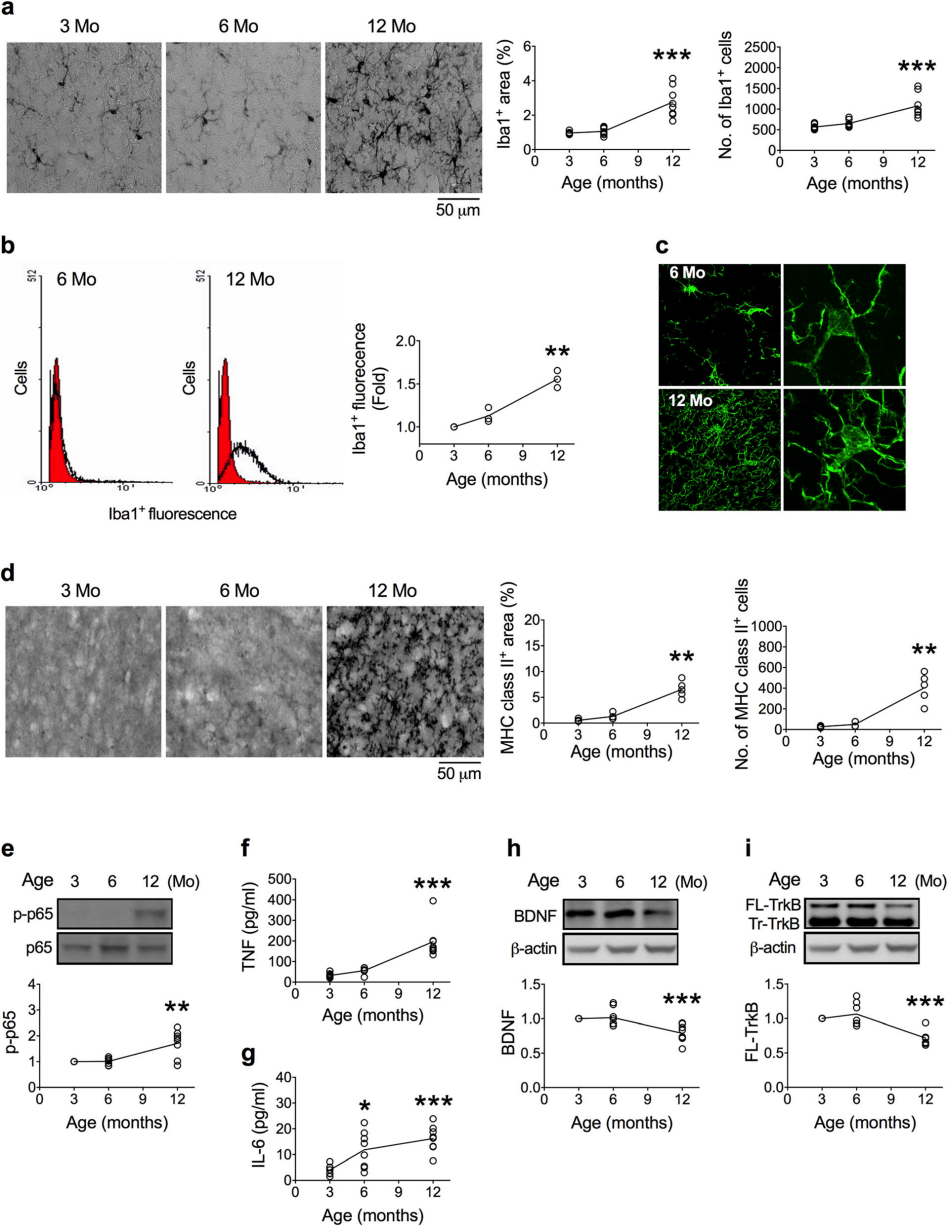
Temporal profiles of BDNF, TrkB, and microglial activation in the substantia nigra during aging. **a** Representative immunohistochemical images show Iba1^+^ cells in the SN of 3-, 6-, and 12-month-old mice. Quantitative results of Iba1^+^ cell areas and numbers (*n* = 8 in each age group) are shown on the right panels. To control for non-specific binding, Iba1 and MHC class II antibodies were replaced by their respective isotype antibodies. **b** Intensity of Iba1^+^ signal in isolated microglia. The distribution of the intensity of Iba1^+^ cells of 3-month-old mice is shown in red as a reference peak. The intensity of 12-month-old mice was much stronger than that of 3-month-old mice as indicated by the right-shifted distribution of the intensity (*n* = 3). **c** Representative confocal micrographs show the Iba1^+^ cells in the SN of 6- and 12-month-old mice. An enlarged single Iba1^+^ cell is both shown on the right panel. **d** Representative immunohistochemical images show MHC class II^+^ cells in the SN of 3-, 6-, and 12-month-old mice. Quantitative results of MHC class II^+^ cell areas and numbers (*n* = 5 in each age group) are shown on the right panels. **e**–**i** Levels of p-p65, TNF, IL-6, BDNF, and FL-TrkB (*n* = 8 in each age group). To control for non-specific binding, Iba1 and MHC class II antibodies were replaced by their respective isotype antibodies. Kruskal-Wallis test: *p < 0.05, **p < 0.01, ***p < 0.001 versus 3-month-old mice. Data are presented as mean ± S.D.

Detail characterization of the SN showed that the areas and numbers of Iba1^+^ cells in the SN were similar in 3- and 6-month-old mice but increased in 12-month-old mice (Fig. 1a). Compared with 3-month-old mice, Iba1^+^ intensities of isolated mesencephalic primary microglia were significantly higher in 12-month-old mice (Fig. 1b). Confocal micrographs revealed that Iba1^+^ cells in the SN of 12-month-old mice had more, but finer (hyper-ramified), processes than those of 6-month-old mice (Fig. 1c).

We utilized another activated microglia marker, MHC class II, to confirm this temporal profile. The areas and numbers of MHC class II^+^ cells significantly increased in the 12-month-old mice (Fig. 1d). Likewise, the levels of p-NF-κB p65 (Fig. 1e), TNF-α (Fig. 1f), and IL-6 (Fig. 1g) in the SN were also higher in the 12-month-old mice. On the contrary, the levels of BDNF (Fig. 1h) and full-length (FL)-TrkB receptor (Fig. 1i) in the SN were significantly decreased in 12-month-old mice. The levels of truncated (Tr)-TrkB did not change in mice up to 12 months old (data not shown).

**Fig. 2:**
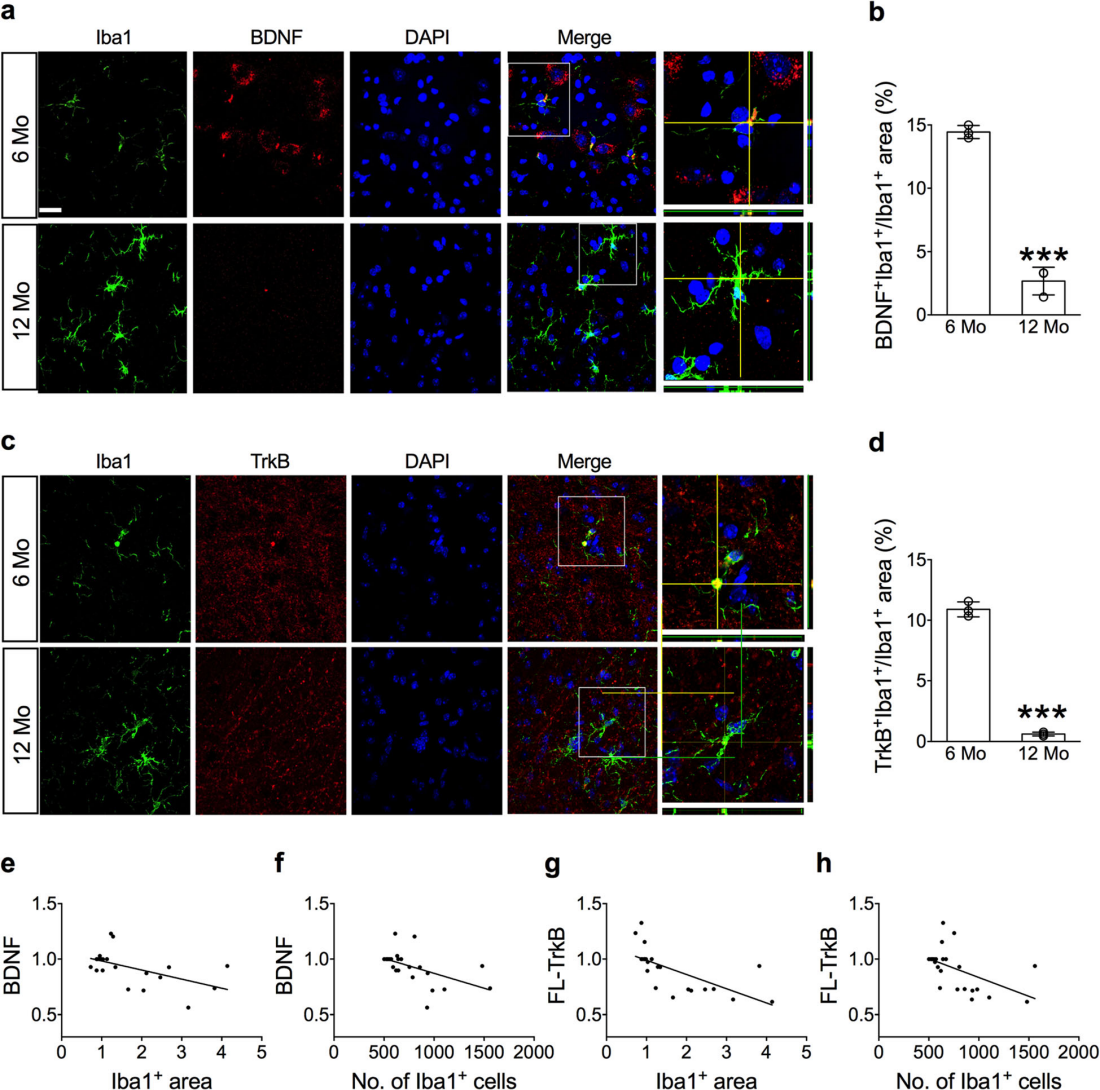
Downregulation of BDNF and TrkB in aged microglial cells. **a**–**b** Representative dual immunofluorescent images show the distribution of BDNF (**a**) and TrkB (**c**) in the Iba1^+^ cells in the SN of 6- and 12-month-old mice. Scale bar: 10 μm. The percent areas of BDNF^+^Iba1^+^/Iba1^+^ (**b**) and TrkB^+^Iba1^+^/Iba1^+^ (**d**) in the SN of 6- and 12-month-old mice. BDNF^+^ areas were detected from 31 cells in three 6-month-old mice and 56 cells in three 12-month-old mice; TrkB^+^ areas were obtained from 35 cells in three 6-month-old mice and 73 cells in three 12-month-old mice. ***p < 0.001 versus 6-month-old mice. **e**–**h** Correlations (Pearson) between the levels of BDNF and TrkB, and the area and number of Iba1^+^ cells (*n* = 24)

Dual immunofluorescence staining showed that the BDNF^+^ signals were profusely expressed in the Iba1^+^ cells of 6-month-old mice but were far fewer expressed in the Iba1^+^ cells of the 12-month-old mice (Fig. 2a). The TrkB^+^ signals in the Iba1^+^ cells also became less intense as the age of the mice increased from 6 to 12 months (Fig. 2c). Specifically, the BDNF^+^ areas (Fig. 2b) and TrkB^+^ areas (Fig. 2d) within the Iba1^+^ cells of the 12-month-old mice were much smaller than those of the 6-month-old mice. The association between BDNF and microglial activation was evaluated using the Pearson correlation. The areas (*r* = − 0.55, *p* = 0.005) (Fig. 2e) and numbers (*r* = − 0.52, *p* = 0.009) (Fig. 2f) of Iba1^+^ cells were negatively correlated with BDNF levels in the SN. Similarly, the areas (*r* = − 0.67, *p* < 0.001) (Fig. 2g) and numbers (*r* = − 0.52, *p* = 0.010) (Fig. 2h) of Iba1^+^ cells were negatively correlated with the levels of FL-TrkB in the SN.

### Long-term supplement of BDNF inhibits microglial activation in aged mice while blocking of BDNF-TrkB signaling induces microglial activation in young mice

**Fig. 3:**
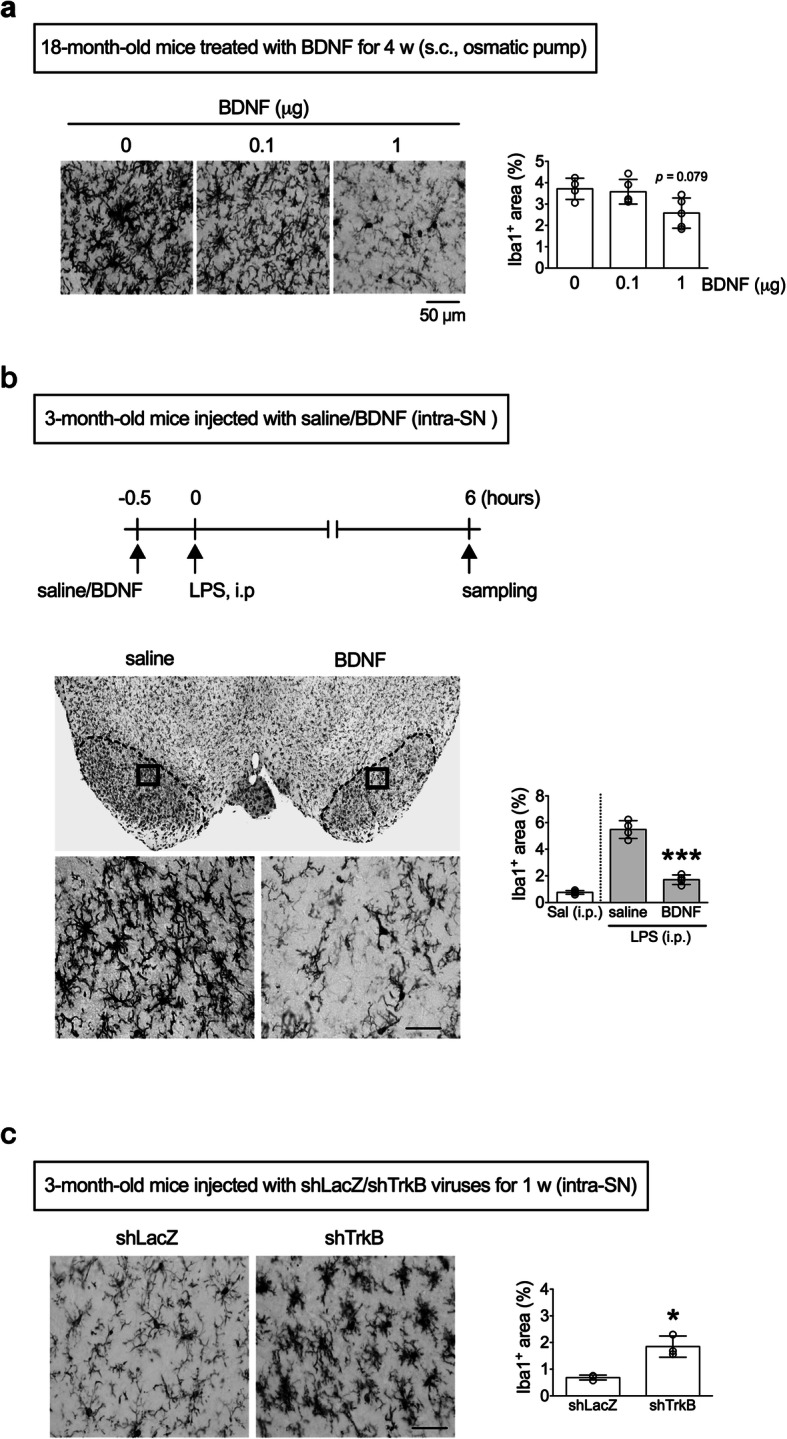
Effect of BDNF on microglial activation in the substantia nigra of mice. **a** Areas of Iba1^+^ cells in the SN of 18-month-old mice that were injected (osmotic pump) with different doses of BDNF (s.c.) for 1 month. Kruskal-Wallis test: *p* = 0.079 versus Saline (0 μg) treated mice (0 μg: *n* = 4; 0.1 μg and 1 μg: *n* = 5). **b** Iba1^+^ cells in the SN of 3-month-old mice 6 h after an intraperitoneal LPS (0.15 mg/kg of body weight) injection. Thirty minutes before the LPS injection, the right SNs were injected with BDNF (1 μl), and the left SNs with an equal amount of saline. The middle panels are enlargements of the boxes in the top panels. Quantitative data for a fixed region (0.049 mm^2^) immediately next to the injection site are shown in the bottom panels. ***p < 0.001 versus the saline injection side of the LPS treatment group (*n* = 4). Sal (i.p.): a group of 3-month-old mice given an intraperitoneal injection of saline, but no BDNF injection. The Sal (i.p.) group was a reference group and was not included in the statistical analysis. **c** Representative immunohistochemical micrographs show Iba1^+^ cells in the SN one week after a single injection of shTrkB to their right SNs and shLacZ viruses to the left SNs of 3-month-old mice. Scale bar, 20 μm. *p < 0.05 versus shLacZ injection side (*n* = 3). Data are presented as mean ± S.D

It has been demonstrated that peripheral BDNF can readily cross the blood-brain barrier [20, 21], and chronic, subcutaneous perfusion of BDNF can affect brain functions [22]. To examine if BDNF can reverse aging-associated microglial activation, we subcutaneously perfused 18-month-old male mice with two different doses of BDNF for 1 month. The results showed that 1 μg, but not 0.1 μg, of BDNF supplements modestly reduced the areas of Iba1^+^ cells in the SN of 18-month-old mice (Fig. 3a).

The effect of BDNF on microglial activation was examined in a peripheral LPS injection model [12]. Thirty minutes before 3-month-old mice were injected (i.p.) with LPS (0.15 mg/kg), their right SNs were perfused with 1 μl of BDNF (0.1 μg/μl) each, while their left counterparts were injected with an equal volume of saline. In this study, the degrees of microglial activation were only determined in the sampled sections nearby the injection. The results showed that systemic LPS injection increased areas of Iba1^+^ in the SN, which were attenuated by local infusion of BDNF (Fig. 3b). To examine whether the effect of BDNF on microglial activation is a global or region-specific phenomenon, we repeated the experiments in the striatum and hippocampus. A similar antimicroglial activation effect of BDNF was also evident in these two regions (Fig. S2a).

To investigate the endogenous BDNF-TrkB signaling on microglial activation, lentiviruses with shTrkB were injected into the right SNs of 3-month-old mice, and their left counterparts were injected with shLacZ lentiviruses. One week later, levels of TrkB in the right SN decreased, while the Iba1^+^ cells enlarged in the vicinity of the shTrkB injection sites (Fig. 3c). Lentiviral knockdown of local TrkB also caused increases of Iba1^+^ area in the striatum and hippocampus (Fig. S2b).

### BDNF inhibits microglial activation in vitro

**Fig. 4.**
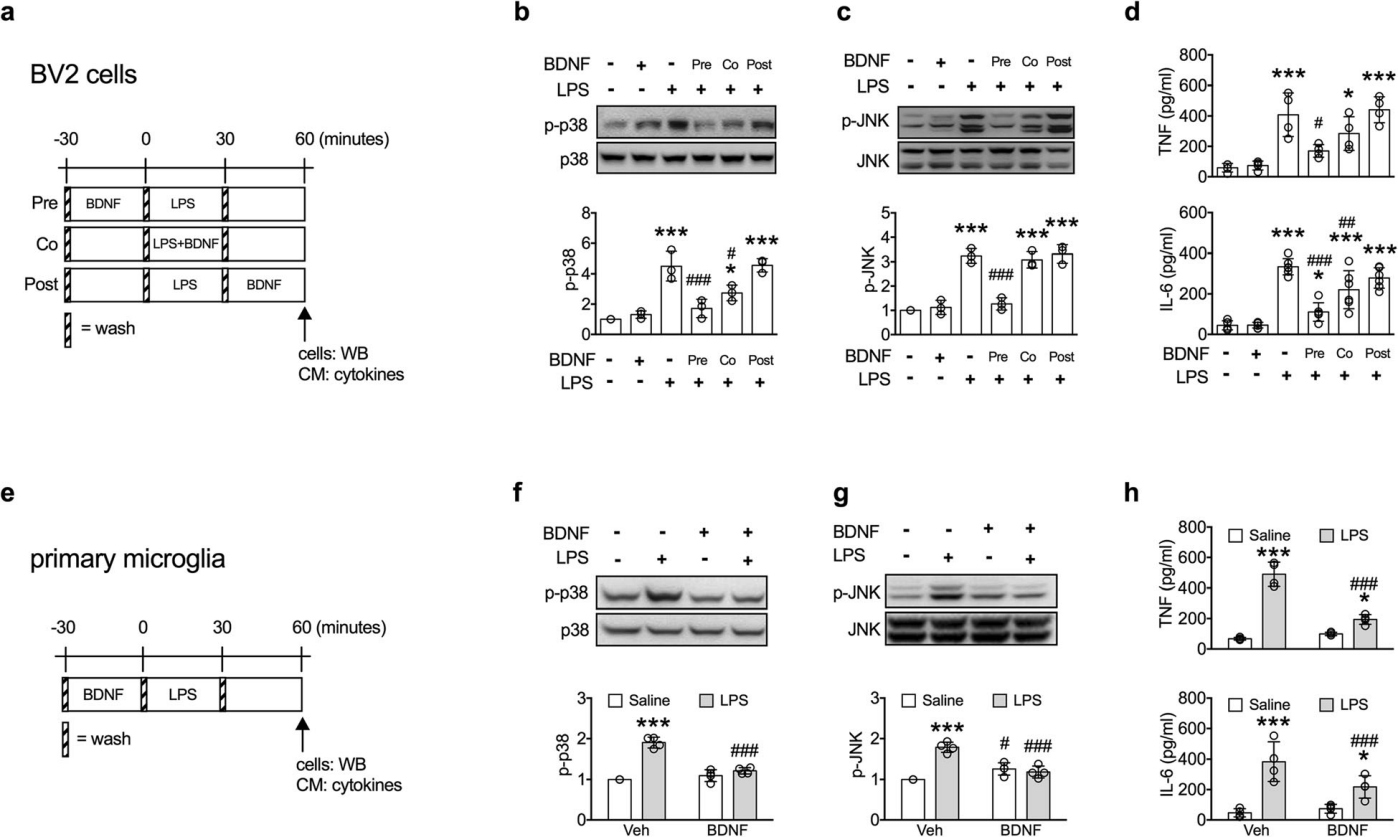
LPS-induced microglial activation was blocked by BDNF pre-treatment. **a–d** BV2 microglial cells. **a** The experimental timeline. **b**, **c** Levels of p-p38 (*n* = 3) and p-JNK (*n* = 3) in the BV2 cells. **d** Levels of TNF (*n* = 4) and IL-6 (*n* = 6) in conditioned media. *p < 0.05, ***p < 0.001 versus BDNF(-)LPS(-) group; ^#^*p* < 0.05, ^##^*p* < 0.01, ^###^*p* < 0.001 versus BDNF(-)LPS(+) group. **e**–**h** Purified primary microglial cells. **e** The experimental timeline. **f**, **g** Levels of p-p38 (*n* = 4) and p-JNK (*n* = 4) in the primary microglial cells. **h** Levels of TNF (*n* = 4) and IL-6 (*n* = 4) in conditioned media. *p < 0.05, ***p < 0.001 versus respective Saline group; ^#^*p* < 0.05, ^###^*p* < 0.001 versus respective Veh group (*n* = 4). Data are presented as mean ± S.D

To investigate the direct antimicroglial activation effect of BDNF, murine microglial BV2 cells were treated with BDNF 30 min before (pre-treatment), concurrently with (co-treatment), and 30 min after (post-treatment) LPS treatment (Fig. 4a). The LPS-induced elevations of p-p38 (Fig. 4b) and p-JNK (Fig. 4c), two critical kinases in regulating inflammatory responses [23, 24], were effectively inhibited by the pre-treatment of BDNF and to a lesser extent by the co-treatment, but not by the post-treatment of BDNF. The LPS-induced productions of TNF-α and IL-6 (Fig. 4d) were also inhibited by the pre-treatment of BDNF. In isolated mesencephalic primary microglia (Fig. 4e), LPS-induced elevations of p-p38 (Fig. 4f) and p-JNK (Fig. 4g), and productions of TNF-α and IL-6 (Fig. 4h) were also inhibited by BDNF pre-treatment.

We also evaluated the effects of different neurotrophic factors (i.e., glial cell-derived neurotrophic factor (GDNF) and insulin-like growth factor (IGF)-1) on LPS-induced microglial activation. Pre-treatment of GDNF inhibited LPS-induced elevations of p-p38 and IL-6 levels, but not p-JNK or TNF-α levels in BV2 cells (Fig. S3). Only LPS-induced elevation of p-p38, but not p-JNK, TNF-α, or IL-6, was inhibited in insulin-like growth factor-1 (IGF-1)-pretreated BV2 cells (Fig. S3).

**Fig. 5:**
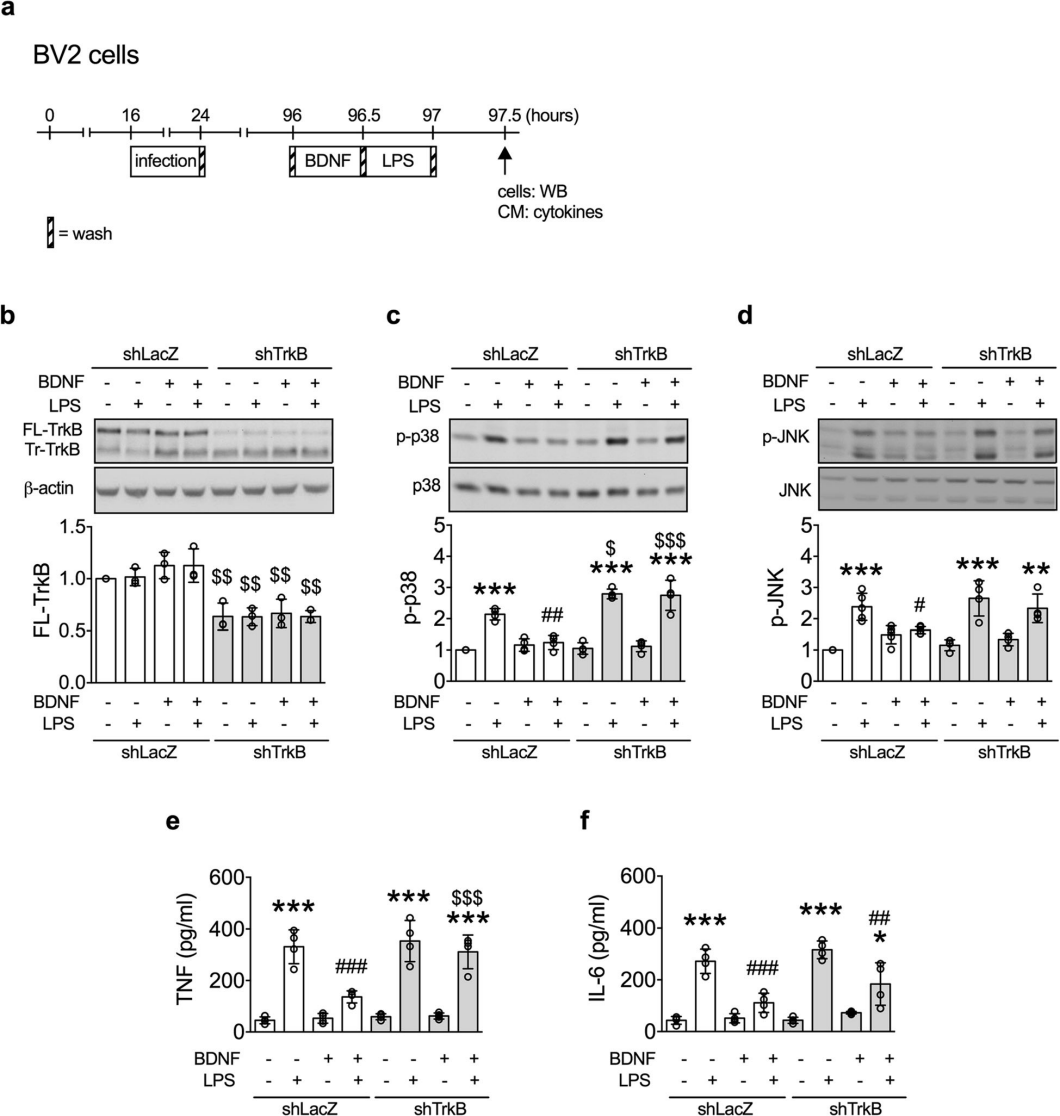
Downregulation of TrkB reversed LPS-induced microglial activation in BDNF-treated BV2 cells. **a** The experimental timeline. **b**–**d** Levels of TrkB (*n* = 3), p-p38 (*n* = 4), and p-JNK (*n* = 4–5) in the BV2 cells. **e**, **f** Levels of TNF (*n* = 4) and IL-6 (*n* = 4) in the conditioned media of BV2 cells. Bonferroni post-hoc test: ^$^*p* < 0.05, ^$$^*p* < 0.01, ^$$$^*p* < 0.001: shTrkB versus respective shLacZ group.*p < 0.05, **p < 0.01, ***p < 0.001: LPS(+) versus respective LPS(-) group; ^#^*p* < 0.05, ^##^*p* < 0.01, ^###^*p* < 0.001: BDNF(+) versus respective BDNF(-) group. Data are presented as mean ± S.D

The antimicroglial activation effect of BDNF signaling was validated using shTrkB (Fig. 5a). In shTrkB-treated BV2 cells, levels of FL-TrkB were decreased (Fig. 5b), but levels of Tr-TrkB did not change. The inhibitory effects of BDNF on LPS-induced activations of p38 (Fig. 5c) and JNK (Fig. 5d) and productions of TNF-α (Fig. 5e) and IL-6 (Fig. 5f) were blocked by the downregulation of TrkB.

### Inhibition of p38-NF-κB and activation of Erk-CREB are required for antimicroglial activation of BDNF

**Fig. 6.**
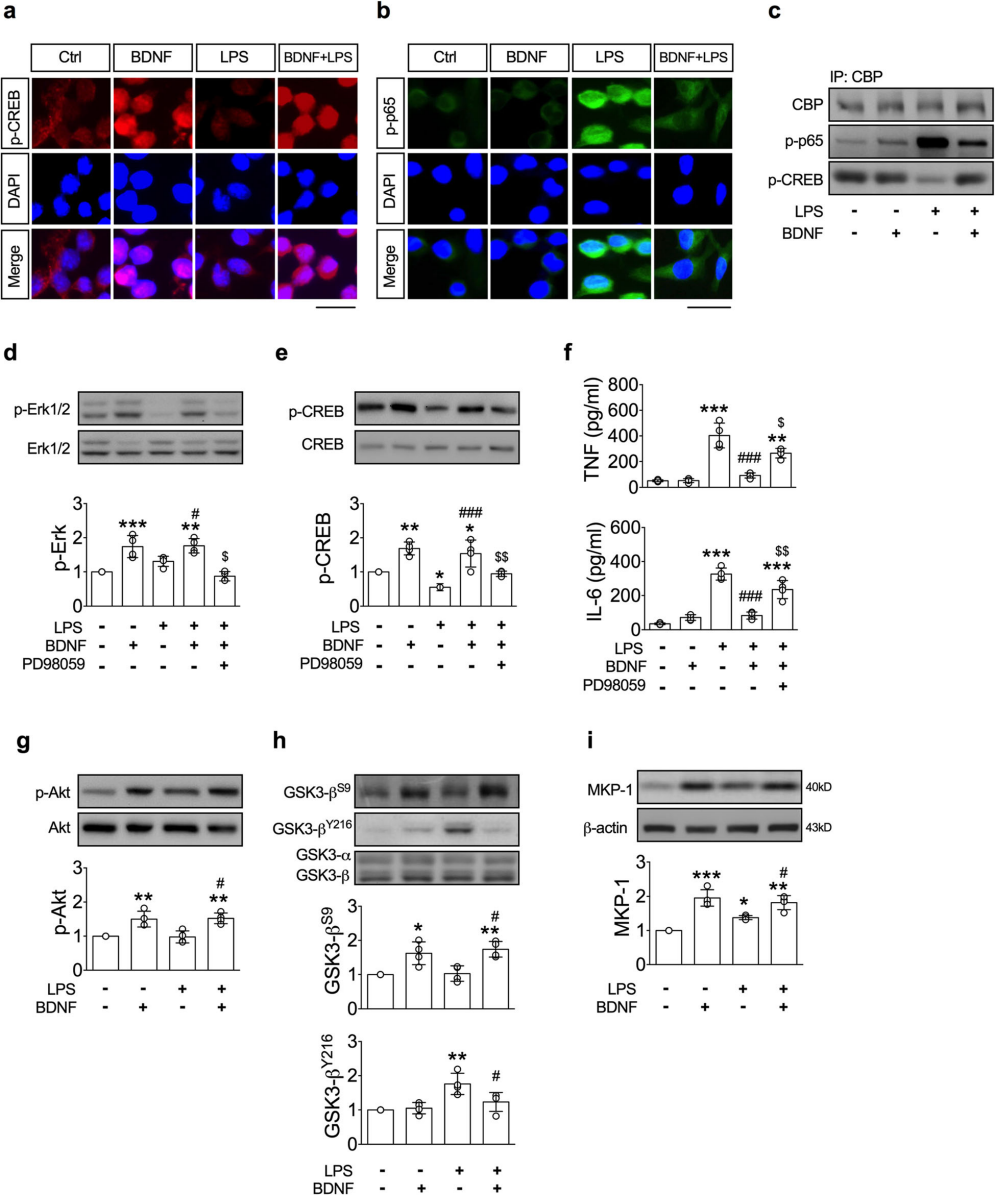
BDNF inhibited microglial activation via upregulation of Erk-CREB signaling. **a**, **b** Representative immunofluorescent images show the distribution of p-CREB and p-p65 in the BV-2 cells. Scale bar, 10 μm. **c** Interaction of CBP with p-CREB and p-p65, assessed by immunoprecipitation of CBP followed by immunoblot for p-CREB and p-p65. **d**, **e** Levels of p-Erk (*n* = 4) and p-CREB (*n* = 4) in the BV2 cells. **f** Levels of TNF (*n* = 4) and IL-6 (*n* = 4) in conditioned media. *p < 0.05, **p < 0.01, ***p < 0.001 versus LPS(-)BDNF(-)PD98059(-) group; ^#^*p* < 0.05, , ^###^*p* < 0.001 versus LPS(+)BDNF(-)PD98059(-) group; ^$^*P* < 0.05, ^$$^*P* < 0.01 versus LPS(+)BDNF(+)PD98059(-) group. **g**–**i** Levels of p-Akt (*n* = 4), p-GSK3-β (*n* = 4) and MKP-1 (*n* = 4) in the BV2 cells. *p < 0.05, **p < 0.01, ***p < 0.001 versus LPS(-)BDNF(-) group; ^#^*p* < 0.05 versus LPS(+)BDNF(-) group. Data are presented as mean ± S.D

We investigated the involvement of the Erk-CREB pathway in BDNF-induced antimicroglial activation because this pathway represents one of the major downstream pathways of BDNF-TrkB signaling [25, 26]. BDNF increased the immunoreactivities of phosphorylated CREB (p-CREB^S133^) in the nuclei of BV2 cells (Fig. 6a). The BDNF-induced elevation and nuclear localization of p-CREB^S133^ were not affected by LPS (Fig. 6a, BDNF vs. BDNF+LPS). On the other hand, LPS increased the immunoreactivity of p-NF-κB p65 in the nuclei of BV2 cells (Fig. 6b). BDNF pre-treatment (30 min) not only decreased the LPS-induced elevation of p-NF-κB p65, but also inhibited their nuclear retention (Fig. 6b, LPS vs. BDNF+LPS). Because the transcriptional activities of CREB and NF-κB p65 are mediated by their associations with the nuclear coactivator CBP [27, 28], we then examined whether competition for CBP binding by these two transcription factors occurred after the treatments of LPS and BDNF. Immunoprecipitation of CBP revealed that the levels of CBP were not affected by BDNF, LPS, or combined treatments (Fig. 6c). LPS increased the binding between p-NF-κB p65 and CBP but decreased the binding between p-CREB^S133^ and CBP. BDNF reestablished the interaction between p-CREB^S133^ and CBP and decreased the LPS-induced interaction between p-NF-κB p65 and CBP (Fig. 6c).

Since activation of Erk represents an important pathway to increase CREB phosphorylation [25, 26], we then examined the role of Erk-CREB signaling in BDNF-induced antimicroglial activation. LPS alone did not alter the levels of p-Erk, but significantly decreased the levels of p-CREB^S133^ (Fig. 6d, e). However, BDNF-induced activations of Erk and CREB were not affected by LPS (Fig. 6d, e). Inhibition of Erk activation using MEK inhibitor-PD98059 (Fig. 6d) blocked BDNF-induced phosphorylation of CREB (Fig. 6e). PD98059 also blocked the inhibitory effects of BDNF on LPS-induced productions of TNF-α and IL-6 (Fig. 6f).

It has been shown that GSK3-β affects the activity of NF-κB positively, but CREB negatively and favors the productions of proinflammatory cytokines [29, 30]. Akt, an important BDNF-TrkB downstream modulator, can phosphorylate GSK3-β at Ser9 (GSK3-β^S9^) and inhibit its activity [29, 31, 32]. Our results showed that BDNF increased the levels of p-Akt (Fig. 6g) and p-GSK3-β^S9^ (Fig. 6h), but did not affect the levels of phosphorylation of GSK3-β at Tyr216 (GSK3-β^Y216^), an active form of GSK3-β. LPS did not alter the levels of p-Akt or p-GSK3-β^S9^; nor did it alter the BDNF-induced elevations of p-Akt and p-GSK3-β^S9^ (Fig. 6g, h). In contrast, the LPS-induced phosphorylation of GSK3-β^Y216^ was inhibited by BDNF (Fig. 6h). These results suggest that BDNF also suppressed the upstream regulators of NF-κB in BV2 cells.

MKP-1, also known as dual specificity phosphatase-1, plays a crucial role in the deactivation of p38 and JNK [33, 34]. We found that BDNF potently increased the levels of MKP-1 (Fig. 6i). However, LPS also mildly, but significantly, increased the levels of MKP-1 (Fig. 6i). When treating the BV2 cells with BDNF 30 min before LPS, the levels of MKP-1 were higher than those of the LPS alone group resembling the BDNF alone group (Fig. 6i).

### BDNF inhibited microglial activation and dopaminergic neuron death in the SN

**Fig. 7:**
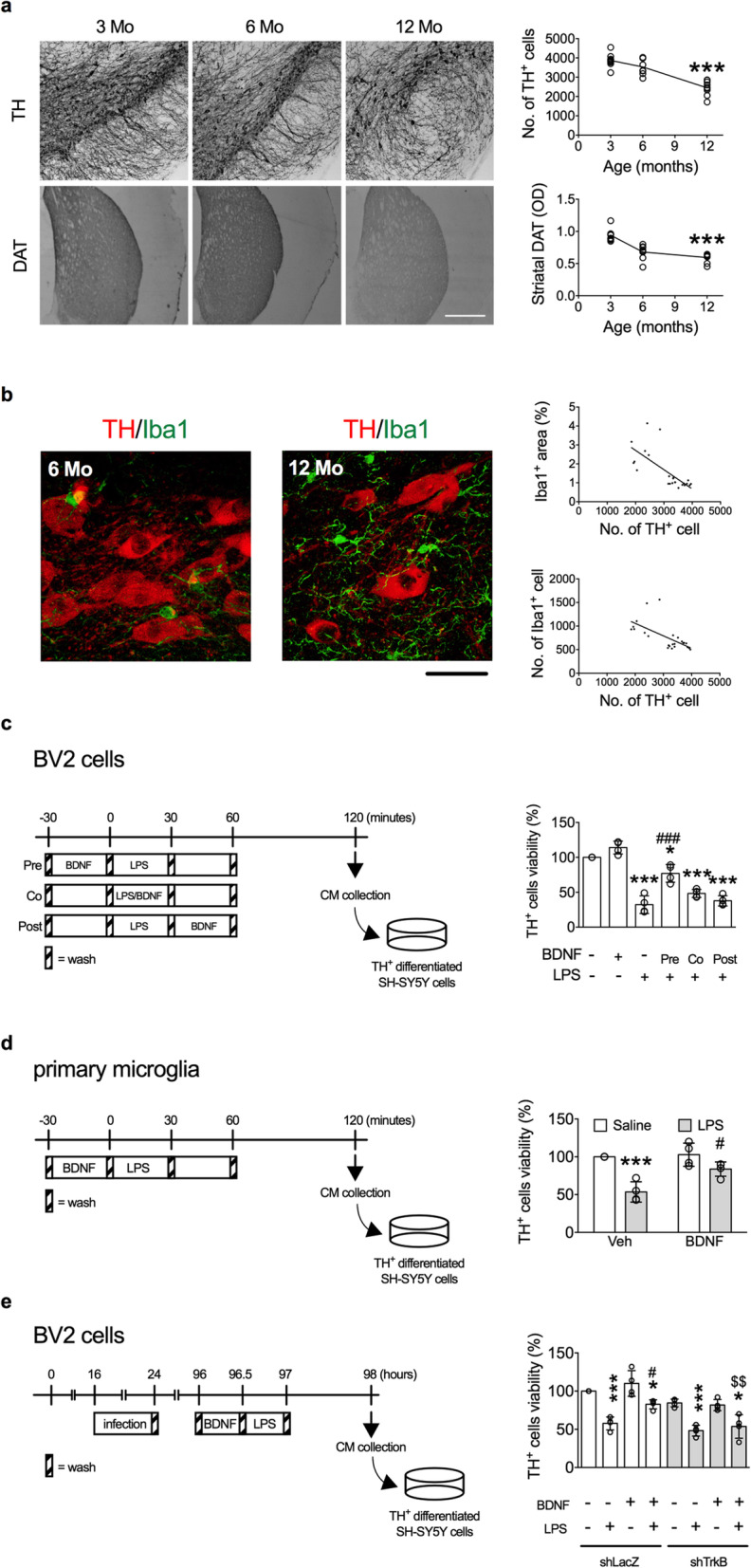
Degrees of microglial activation negatively correlate with the survival of DA neurons in the SN during aging and in vitro. **a** Representative immunohistochemical images show TH^+^ cells in the SN and DAT^+^ signal in the striatum of 3-, 6-, and 12-month-old mice. Quantitative results of numbers of TH^+^ cells and intensities of DAT^+^ signal (*n* = 8 in each age group) are shown on the right panels. To control for non-specific binding, TH and DAT antibodies were replaced by their respective isotype antibodies. **b** Representative confocal micrographs show the Iba1^+^ and TH^+^ cells in the SN of 6- and 12-month-old mice. Correlations (Pearson) between numbers of TH^+^ cells and areas and numbers of Iba1^+^ cells are shown on the right panels (*n* = 24). **c** Viability of TH^+^ cells cultured for 24 h in conditioned media of BV2 cells (*n* = 4). The experimental timeline is shown on the left panel, while the cell survival rate is shown on the right panel. *p < 0.05, ***p < 0.001 versus BDNF(-)LPS(-) group; ^###^*p* < 0.001 versus BDNF(-)LPS(+) group. **d** Viability of TH^+^ cells cultured for 24 h in conditioned media of primary microglia (*n* = 4). The experimental timeline is shown on the left panel, while the cell survival rate is shown on the right panel. ***p < 0.001 versus respective Saline group; ^#^*p* < 0.05 versus respective Veh group (*n* = 4). **e** Viability of TH^+^ cells cultured for 24 h in conditioned media of virus-infected BV2 cells (*n* = 4). The experimental timeline is shown on the left panel, while the cell survival rate is shown on the right panel. Bonferroni post-hoc test: ^$$^*p* < 0.01: shTrkB versus respective shLacZ group. *p < 0.05, ***p < 0.001: LPS(+) versus respective LPS(-) group; ^#^*p* < 0.05: BDNF(+) versus respective BDNF(-) group. Data are presented as mean ± S.D.

The nigrostriatal pathway is critical for movement controls, and loss of DA neurons in the SN is one of the pathological hallmarks of Parkinson’s disease [35]. The precise mechanism for DA neuron loss in the SN during aging remains unclear. It has been demonstrated that degrees of local inflammation and microglial activation are highly correlated to the severities of DA neuron death in the SN [36–38]. We then attempted to address the role of BDNF in microglial activation and DA neuronal loss during aging. The results showed that numbers of TH^+^ DA neurons were decreased as the age increased in the SN of mice (Fig. 7a). Similar changes were also evident in the striatal DAT^+^ signals (Fig. 7a). The association between numbers of DA neurons and degrees of microglial activation was evaluated (Fig. 7b). The areas (*r* = − 0.71, *p* < 0.001) and numbers (*r* = − 0.64, *p* < 0.001) of Iba1^+^ cells were negatively correlated with numbers of DA neurons in the SN (Fig. 7b).

The neurotoxicity of microglia regulated by BDNF was examined by treating microglial conditioned medium to the TH^+^ neurons differentiated from the SH-SY5Y cells. The conditioned media were collected from murine microglial BV2 cells 60 min after the pre-, co-, and post-treatment of BDNF and LPS (Fig. 7c). Our results showed that the LPS-induced neurotoxicities of BV2 cells were inhibited by BDNF pre-treatment (Fig. 7c). Likewise, LPS-induced neurotoxicities of isolated primary microglia were also attenuated by BDNF pre-treatment (Fig. 7d). BDNF inhibition of LPS-induced microglial neurotoxicity was also eliminated in shTrkB-treated BV2 cells (Fig. 7e).

## Discussion

We compared the degrees of microglial activation and BDNF-TrkB signaling in mice of different ages and found that the level of BDNF-TrkB was reduced and the degree of microglial activation was enhanced. In addition to neurons, BDNF and TrkB were richly expressed in microglia, and their levels were negatively correlated with the degree of microglial activation. In mice, an exogenous supplement of BDNF alone was sufficient to counteract the aging-related and LPS-induced microglial activation. Injecting shTrkB viruses into various brain regions of young mice consistently induced local microglial activation. These findings further suggest the involvement of neurotrophic tyrosine kinases and their downstream factors in microglial activation.

Chronic perfusion of recombinant BDNF to the periphery could decrease microglial activation in the central nervous system. BDNF can cross the blood-brain barrier into the brain parenchyma [20], and peripheral BDNF perfusion has been shown to increase the phosphorylation of CREB in the brain [39]. Thus, systemic injection of BDNF may constitute a useful approach to inhibit microglial activation. Together, these results suggest that BDNF-TrkB signaling plays a critical role in modulating microglial activation.

By using cultured microglial cell models, we demonstrated that BDNF inhibited LPS-induced microglial activation and related responses, in agreement with previous observations [40–42]. The antimicroglial activation effect of BDNF could be eliminated by shTrkB treatment. Two different neurotrophic factors, GDNF and IGF-1, also induced similar, but weaker, antimicroglial activation responses compared to those induced by BDNF. These findings suggest the involvement of neurotrophic tyrosine kinases and their downstream factors in microglial activation.

Interestingly, Zhang et al. have demonstrated an autocrine effect of microglial BDNF resulting in a prolonged microglial activation [43]. Using ATP as the activator, they showed that ATP activated BV2 microglial cells by enhancing migration and TNF release. Moreover, ATP also increased BDNF synthesis in the cells. Microglial activation was inhibited after the sequestration of endogenous BDNF [43]. In a drug-induced cystitis animal study, the authors showed that systemic intraperitoneal injection of cyclophosphamide induced upregulation of BDNF-TrkB signaling, activation of astrocytes and microglia, and several proinflammatory cytokines in the spinal dorsal horn of rats [44]. Intraperitoneal injection of the TrkB receptor antagonist inhibited mechanical allodynia and activation of astrocytes and microglia, while intrathecal injection of BDNF promoted mechanical allodynia and activation of astrocytes and microglia, as well as proinflammatory cytokines in the spinal dorsal horn [44]. Thus, BDNF may differentially regulate microglial responses depending on the types of inflammatory stimulators and nearby cellular compositions (e.g., central and peripheral nervous system).

We showed that BDNF treatment increased levels of p-Erk1/2 and p-CREB, while inhibition of Erk activity decreased levels of p-CREB and blocked the antimicroglial activation effect of BDNF. In addition to the elevation of CREB phosphorylation, BDNF also increased nuclear localization of CREB, reduced the binding between NF-κB and CBP, and inhibited proinflammatory cytokine production. It has been demonstrated that activated CREB could inhibit NF-κB activity through competition for limited amounts of CBP [27]. Therefore, the BDNF-induced antimicroglial activation responses can be achieved by the antagonizing effect of activated CREB on NF-κB. Furthermore, activated CREB and Erk have been shown to induce a positive feed-forward production of BDNF [45] and upregulate the expression of MKP-1 [27, 46]. Therefore, the BDNF-induced upregulation of MKP-1 may be due to an activation of the Erk-CREB pathway.

It is noteworthy that the BV2 cell model used to characterize the signaling pathways involved in the BDNF-induced antimicroglial activation may not authentically recapture the process of microglial activation occurring in the aging brains. We have attempted to isolate microglia from mice of different ages by following a published protocol [47]. However, we failed to collect sufficient numbers of microglial cells that met the qualification for subsequent analyses. This was especially true in aged mice. To validate our findings, it is necessary to use more advanced techniques in the future to monitor microglia during aging.

In this study, we demonstrated that BDNF could inhibit microglial activation at multiple steps. First, BDNF inhibited the LPS-induced activation of p38 and JNK. A possible mechanism for the inhibitory effect of BDNF might be due to an upregulation of MKP-1, which dephosphorylated p38 and JNK. Although LPS treatment also increased the expression of MKP-1, the low levels of MKP-1 were not sufficient to suppress the LPS-induced activation of p38 and JNK. Second, BDNF activated Akt, which then inactivated the activity of GSK-3. Activated GSK-3 could phosphorylate NF-κB [29, 30]. Inhibition of GSK-3 diminished the LPS-induced activation of NF-κB and the production of proinflammatory cytokines [29]. Furthermore, activated GSK-3β attenuated growth factor-stimulated CREB DNA activity, while the reduction of GSK3β activity (GSK3-β^S9^) increased CREB DNA binding activity [48, 49]. Thus, inhibition of GSK-3β might also contribute to the BDNF-induced activation of CREB. Third, BDNF activated the Erk-CREB pathway, which was necessary for the antimicroglial activation effect of BDNF.

The precise mechanism for DA neuron loss in the SN is unclear. In addition to aging, the most important risk factor for idiopathic PD [49], inflammation is also closely related to DA neuron death in the SN [36–38]. The levels of the proinflammatory cytokines are elevated in the cerebrospinal fluid, serum, striatum, and SN of PD patients [38]. Using positron emission tomography scan with radiotracers for activated microglia and DAT, a negative correlation between these two markers in the dopaminergic nigrostriatal system has been reported in early PD patients [50], suggesting that microglia are activated early in the disease. Our results support the notion that activated microglia induce DA neuron loss and that BDNF may have the potential to inhibit the aging-related DA neuron loss in the SN by its antimicroglial action. In agreement with our findings, it has been demonstrated that levels of BDNF and TrkB are reduced in the nigrostriatal system of PD patients [51–53] and aged rats [54]. Although the SN may not be the most affected brain regions of age-related decline of BDNF, local microglia are highly sensitive to inflammatory stimulation [10, 11]. Therefore, decreases of BDNF in the SN could be a major contributor to the age-related increases of microglial activation and DA neuron loss.

## Conclusions

In summary, we demonstrated a critical role of BDNF-TrkB signaling in regulating microglia inflammation responses. Decreases of BDNF-TrkB signaling in microglia during aging are associated with their activation in the SN, while systemic delivery of BDNF reversed aging-related microglial activation. In a cultured microglial cell line, BDNF blocked LPS-induced microglial activation. Activation of the TrkB/Erk/CREB pathway was necessary for the BDNF-induced antimicroglial activation response. Although more studies are needed to validate the in vitro findings in the aging brain, this study sheds light on the potential of BDNF for controlling microglial activation and the inflammation-associated neurodegenerative diseases.

## Supplementary information

**Additional file 1:** Fig. S1.

**Additional file 2:** Fig. S2.

**Additional file 3:** Fig. S3.

## Data Availability

The datasets used and/or analyzed in this study are available from the corresponding author on reasonable request.

## Abbreviations

BDNF: Brain-derived neurotrophic factor CBP CREB-binding protein DA Dopaminergic DAT Dopamine transporter DMEM/F12 Dulbecco’s modified Eagle’s medium/F12 FBS Fetal bovine serum FL Full-length GDNF Glial cell-derived neurotrophic factor GSK3 Glycogen synthase kinase 3 Iba1 Ionized calcium-binding adapter molecule-1 IGF-1 Insulin-like growth factor-1 LPS Lipopolysaccharide MHC Major histocompatibility complex MKP-1 Mitogen-activated protein kinase phosphatase-1 PBS Phosphate-buffered saline SN Substantia nigra TH tyrosine hydroxylase Tr Truncated

## References

[1] Perry VH, Holmes C (2014). Microglial priming in neurodegenerative disease. Nat Rev Neurol.

[2] Nimmerjahn A, Kirchhoff F, Helmchen F (2005). Resting microglial cells are highly dynamic surveillants of brain parenchyma in vivo. Science.

[3] Hanisch UK, Kettenmann H (2007). Microglia: active sensor and versatile effector cells in the normal and pathologic brain. Nat Neurosci.

[4] Ghosh A, Carnahan J, Greenberg ME (1994). Requirement for BDNF in activity-dependent survival of cortical neurons. Science.

[5] Numakawa T, Suzuki S, Kumamaru E, Adachi N, Richards M, Kunugi H (2010). BDNF function and intracellular signaling in neurons. Histol Histopathol.

[6] Jiang Y, Wei N, Lu T, Zhu J, Xu G, Liu X (2011). Intranasal brain-derived neurotrophic factor protects brain from ischemic insult via modulating local inflammation in rats. Neuroscience.

[7] Jiang Y, Wei N, Zhu J, Lu T, Chen Z, Xu G, Liu X (2010). Effects of brain-derived neurotrophic factor on local inflammation in experimental stroke of rat. Mediators Inflamm.

[8] Makar TK, Trisler D, Sura KT, Sultana S, Patel N, Bever CT (2008). Brain derived neurotrophic factor treatment reduces inflammation and apoptosis in experimental allergic encephalomyelitis. J Neurol Sci.

[9] Bovolenta R, Zucchini S, Paradiso B, Rodi D, Merigo F, Navarro Mora G, Osculati F, Berto E, Marconi P, Marzola A (2010). Hippocampal FGF-2 and BDNF overexpression attenuates epileptogenesis-associated neuroinflammation and reduces spontaneous recurrent seizures. J Neuroinflammation.

[10] Yang TT, Lin C, Hsu CT, Wang TF, Ke FY, Kuo YM (2013). Differential distribution and activation of microglia in the brain of male C57BL/6 J mice. Brain Struct Funct.

[11] Ji KA, Eu MY, Kang SH, Gwag BJ, Jou I, Joe EH (2008). Differential neutrophil infiltration contributes to regional differences in brain inflammation in the substantia nigra pars compacta and cortex. Glia.

[12] Qin L, Wu X, Block ML, Liu Y, Breese GR, Hong JS, Knapp DJ, Crews FT (2007). Systemic LPS causes chronic neuroinflammation and progressive neurodegeneration. Glia.

[13] Wu SY, Chen YW, Tsai SF, Wu SN, Shih YH, Jiang-Shieh YF, Yang TT, Kuo YM: Estrogen ameliorates microglial activation by inhibiting the Kir2.1 inward-rectifier K(+) channel. Sci Rep 2016, 6:22864. 10.1038/srep22864 PMC4785403 26960267

[14] Guneykaya D, Ivanov A, Hernandez DP, Haage V, Wojtas B, Meyer N, Maricos M, Jordan P, Buonfiglioli A, Gielniewski B (2018). Transcriptional and translational differences of microglia from male and female brains. Cell Rep.

[15] Kodama L, Gan L (2019). Do microglial sex differences contribute to sex differences in neurodegenerative diseases?. Trends Mol Med.

[16] Liu Z, Chen HQ, Huang Y, Qiu YH, Peng YP (2016). Transforming growth factor-beta1 acts via TbetaR-I on microglia to protect against MPP(+)-induced dopaminergic neuronal loss. Brain Behav Immun.

[17] Xie HR, Hu LS, Li GY (2010). SH-SY5Y human neuroblastoma cell line: in vitro cell model of dopaminergic neurons in Parkinson's disease. Chin Med J (Engl).

[18] Hancock MB (1984). Visualization of peptide-immunoreactive processes on serotonin-immunoreactive cells using two-color immunoperoxidase staining. J Histochem Cytochem.

[19] de Haas AH, Boddeke HW, Brouwer N, Biber K (2007). Optimized isolation enables ex vivo analysis of microglia from various central nervous system regions. Glia.

[20] Poduslo JF, Curran GL (1996). Permeability at the blood-brain and blood-nerve barriers of the neurotrophic factors: NGF, CNTF, NT-3, BDNF. Brain Res Mol Brain Res.

[21] Pan W, Banks WA, Fasold MB, Bluth J, Kastin AJ (1998). Transport of brain-derived neurotrophic factor across the blood-brain barrier. Neuropharmacology.

[22] Schmidt HD, Duman RS (2010). Peripheral BDNF produces antidepressant-like effects in cellular and behavioral models. Neuropsychopharmacology.

[23] Saccani S, Pantano S, Natoli G (2002). p38-Dependent marking of inflammatory genes for increased NF-kappa B recruitment. Nat Immunol.

[24] Dong C, Davis RJ, Flavell RA (2002). MAP kinases in the immune response. Annu Rev Immunol.

[25] Pizzorusso T, Ratto GM, Putignano E, Maffei L (2000). Brain-derived neurotrophic factor causes cAMP response element-binding protein phosphorylation in absence of calcium increases in slices and cultured neurons from rat visual cortex. J Neurosci.

[26] Ying SW, Futter M, Rosenblum K, Webber MJ, Hunt SP, Bliss TV, Bramham CR (2002). Brain-derived neurotrophic factor induces long-term potentiation in intact adult hippocampus: requirement for ERK activation coupled to CREB and upregulation of Arc synthesis. J Neurosci.

[27] Wen AY, Sakamoto KM, Miller LS (2010). The role of the transcription factor CREB in immune function. J Immunol.

[28] Parry GC, Mackman N (1997). Role of cyclic AMP response element-binding protein in cyclic AMP inhibition of NF-kappaB-mediated transcription. J Immunol.

[29] Martin M, Rehani K, Jope RS, Michalek SM (2005). Toll-like receptor-mediated cytokine production is differentially regulated by glycogen synthase kinase 3. Nat Immunol.

[30] Hoeflich KP, Luo J, Rubie EA, Tsao MS, Jin O, Woodgett JR (2000). Requirement for glycogen synthase kinase-3beta in cell survival and NF-kappaB activation. Nature.

[31] Cohen P, Frame S (2001). The renaissance of GSK3. Nat Rev Mol Cell Biol.

[32] Cross DA, Alessi DR, Cohen P, Andjelkovich M, Hemmings BA (1995). Inhibition of glycogen synthase kinase-3 by insulin mediated by protein kinase B. Nature.

[33] Owens DM, Keyse SM (2007). Differential regulation of MAP kinase signalling by dual-specificity protein phosphatases. Oncogene.

[34] Chen P, Li J, Barnes J, Kokkonen GC, Lee JC, Liu Y (2002). Restraint of proinflammatory cytokine biosynthesis by mitogen-activated protein kinase phosphatase-1 in lipopolysaccharide-stimulated macrophages. J Immunol.

[35] Samii A, Nutt JG, Ransom BR (2004). Parkinson's disease. Lancet.

[36] Block ML, Hong JS (2005). Microglia and inflammation-mediated neurodegeneration: multiple triggers with a common mechanism. Prog Neurobiol.

[37] Block ML, Zecca L, Hong JS (2007). Microglia-mediated neurotoxicity: uncovering the molecular mechanisms. Nat Rev Neurosci.

[38] Hirsch EC, Hunot S (2009). Neuroinflammation in Parkinson's disease: a target for neuroprotection?. Lancet Neurol.

[39] Giampa C, Montagna E, Dato C, Melone MA, Bernardi G, Fusco FR (2013). Systemic delivery of recombinant brain derived neurotrophic factor (BDNF) in the R6/2 mouse model of Huntington's disease. PLoS One.

[40] Mizoguchi Y, Kato TA, Seki Y, Ohgidani M, Sagata N, Horikawa H, Yamauchi Y, Sato-Kasai M, Hayakawa K, Inoue R (2014). Brain-derived neurotrophic factor (BDNF) induces sustained intracellular Ca2+ elevation through the up-regulation of surface transient receptor potential 3 (TRPC3) channels in rodent microglia. J Biol Chem.

[41] Ye X, Yu L, Zuo D, Zhang L, Zu J, Hu J, Tang J, Bao L, Cui C, Zhang R, Jin G, Zan K, Zhang Z, Yang X, Shi H, Zhang Z, Xiao Q, Liu Y, Xiang J, Zhang X, Cui G (2017). Activated mGluR5 protects BV2 cells against OGD/R induced cytotoxicity by modulating BDNF-TrkB pathway. Neurosci Lett.

[42] Zhou J, Wamg M, Deng D (2020). KLF2 protects BV2 microglial cells against oxygen and glucose deprivation injury by modulating BDNF/TrkB pathway. Gene.

[43] Zhang X, Zeng L, Yu T, Xu Y, Pu S, Du D, Jiang W (2014). Positive feedback loop of autocrine BDNF from microglia causes prolonged microglia activation. Cell Physiol Biochem.

[44] Ding H, Chen J, Su M, Lin Z, Zhan H, Yang F, Li W, Xie J, Huang Y, Liu X (2020). BDNF promotes activation of astrocytes and microglia contributing to neuroinflammation and mechanical allodynia in cyclophosphamide-induced cystitis. J Neuroinflammation.

[45] Larson EB, Graham DL, Arzaga RR, Buzin N, Webb J, Green TA, Bass CE, Neve RL, Terwilliger EF, Nestler EJ, Self DW (2011). Overexpression of CREB in the nucleus accumbens shell increases cocaine reinforcement in self-administering rats. J Neurosci.

[46] Cook SJ, Beltman J, Cadwallader KA, McMahon M, McCormick F (1997). Regulation of mitogen-activated protein kinase phosphatase-1 expression by extracellular signal-related kinase-dependent and Ca2+-dependent signal pathways in Rat-1 cells. J Biol Chem.

[47] Lee JK, Tansey MG (2013). Microglia isolation from adult mouse brain. Methods Mol Biol.

[48] Grimes CA, Jope RS (2001). CREB DNA binding activity is inhibited by glycogen synthase kinase-3 beta and facilitated by lithium. J Neurochem.

[49] Collier TJ, Kanaan NM, Kordower JH (2011). Ageing as a primary risk factor for Parkinson's disease: evidence from studies of non-human primates. Nat Rev Neurosci.

[50] Ouchi Y, Yoshikawa E, Sekine Y, Futatsubashi M, Kanno T, Ogusu T, Torizuka T (2005). Microglial activation and dopamine terminal loss in early Parkinson's disease. Ann Neurol.

[51] Howells DW, Porritt MJ, Wong JY, Batchelor PE, Kalnins R, Hughes AJ, Donnan GA (2000). Reduced BDNF mRNA expression in the Parkinson's disease substantia nigra. Exp Neurol.

[52] Mogi M, Togari A, Kondo T, Mizuno Y, Komure O, Kuno S, Ichinose H, Nagatsu T (1999). Brain-derived growth factor and nerve growth factor concentrations are decreased in the substantia nigra in Parkinson's disease. Neurosci Lett.

[53] Parain K, Murer MG, Yan Q, Faucheux B, Agid Y, Hirsch E, Raisman-Vozari R (1999). Reduced expression of brain-derived neurotrophic factor protein in Parkinson's disease substantia nigra. Neuroreport.

[54] Croll SD, Ip NY, Lindsay RM, Wiegand SJ (1998). Expression of BDNF and trkB as a function of age and cognitive performance. Brain Res.

