# Supplementary figures and images for: BDNF reverses aging-related microglial activation
Source: J Neuroinflammation. 2020 Jul 14;17:210. doi: 10.1186/s12974-020-01887-1 (PMC7362451; doi:10.1186/s12974-020-01887-1)

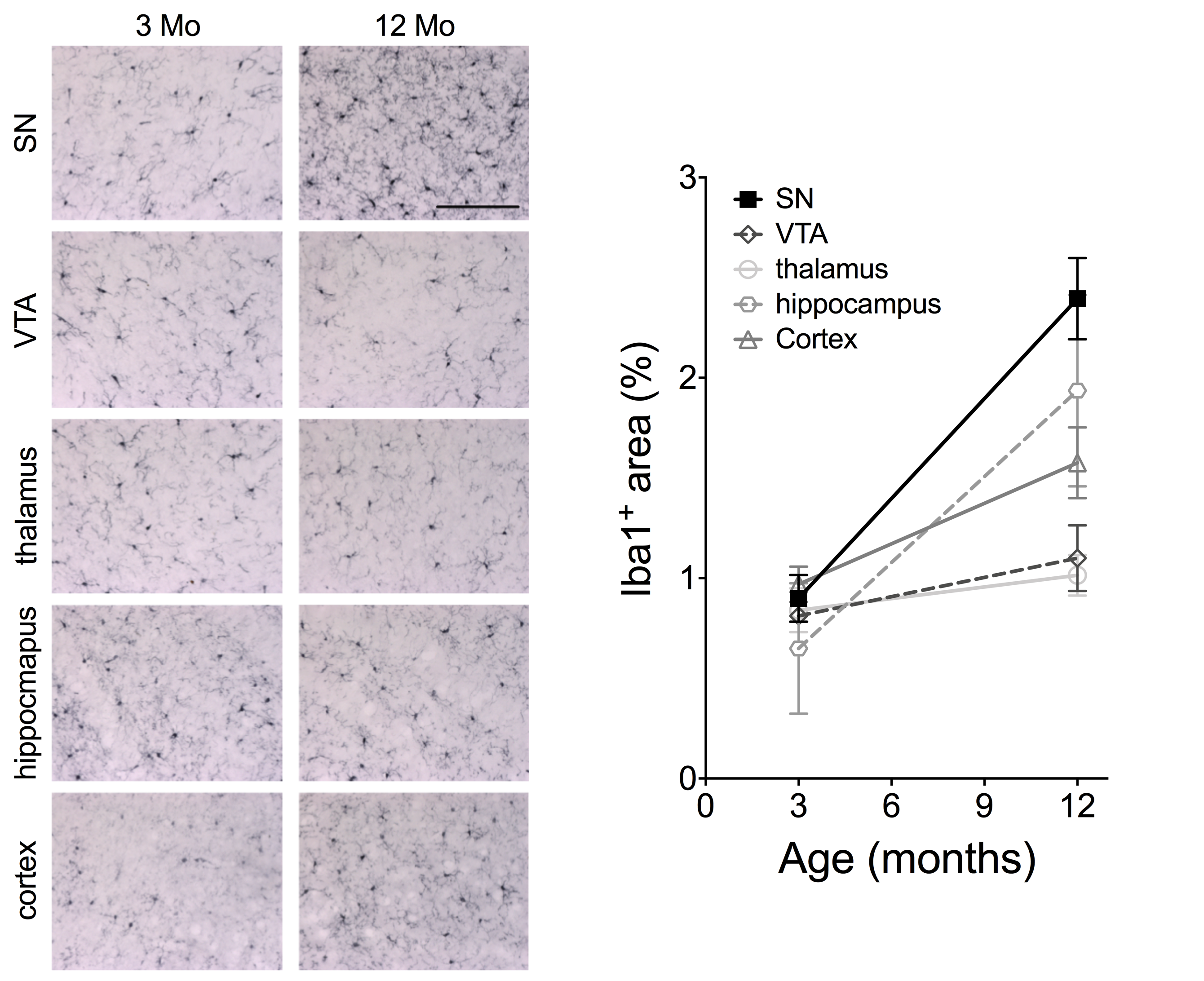

Supplement: Supplementary file 1 — Additional file 1: Fig. S1. [file 12974_2020_1887_MOESM1_ESM.tiff]

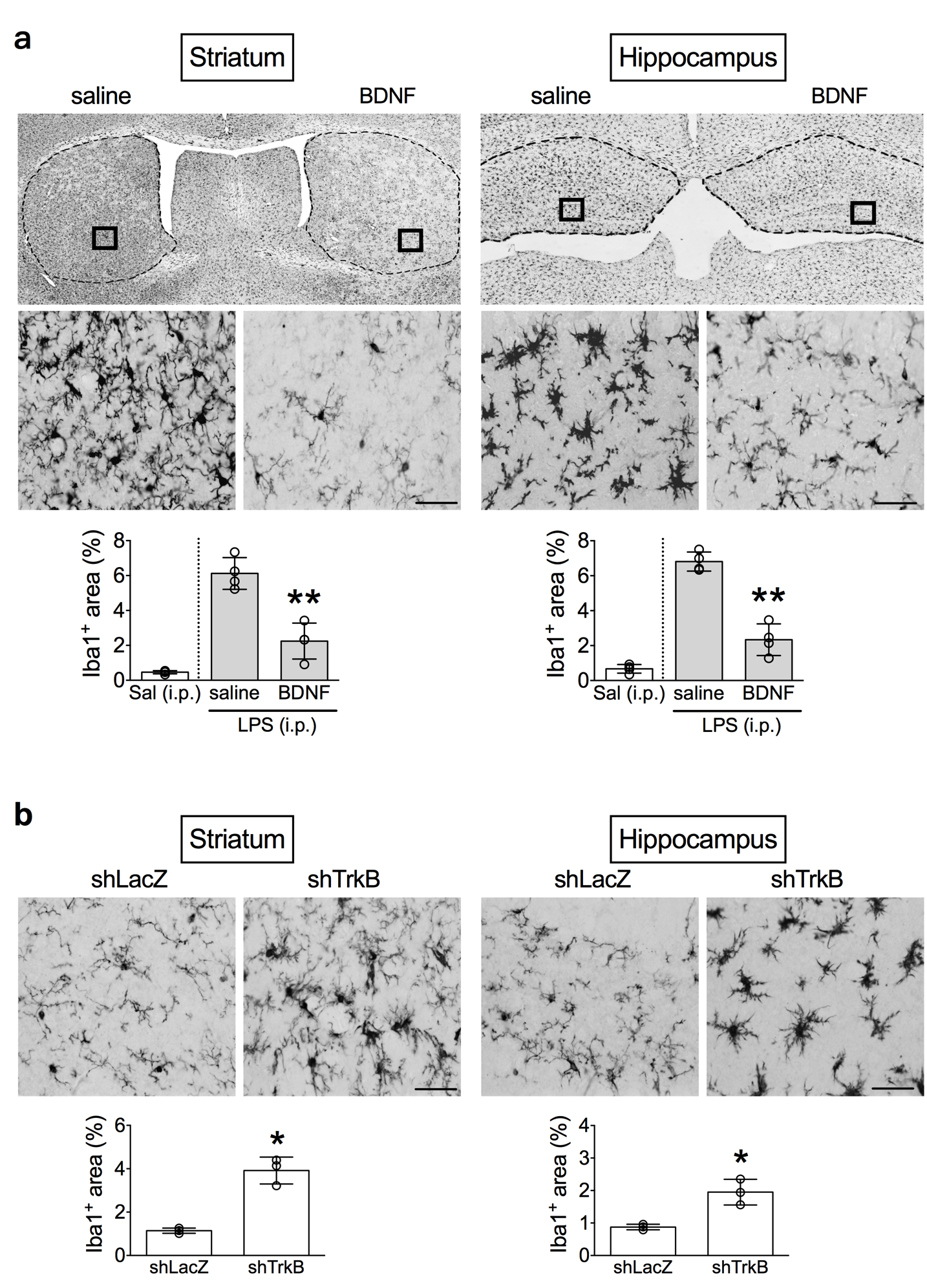

Supplement: Supplementary file 2 — Additional file 2: Fig. S2. [file 12974_2020_1887_MOESM2_ESM.tiff]

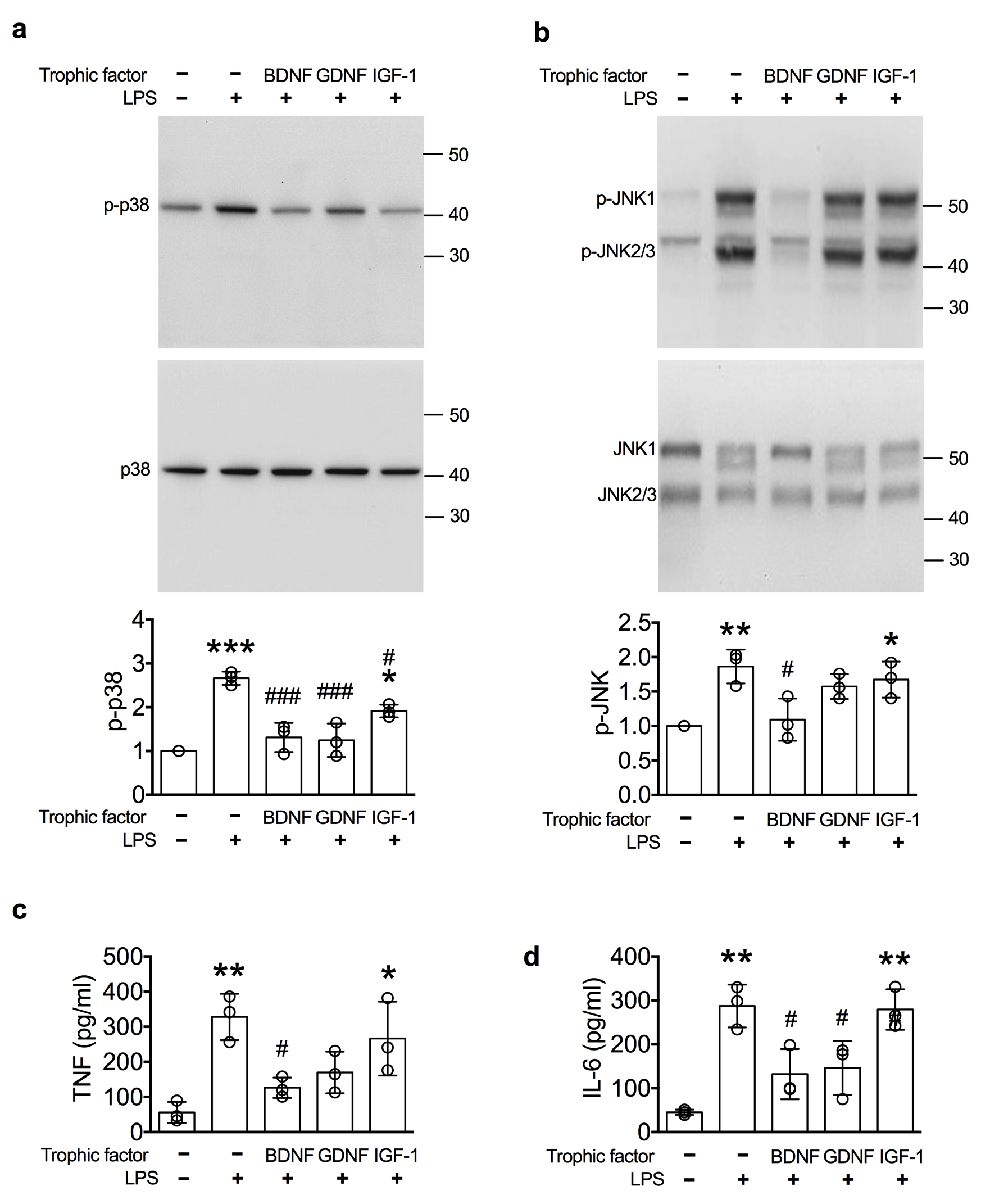

Supplement: Supplementary file 3 — Additional file 3: Fig. S3. [file 12974_2020_1887_MOESM3_ESM.tiff]
